# Cause of death and significant disease found at autopsy

**DOI:** 10.1007/s00428-019-02672-z

**Published:** 2019-11-05

**Authors:** Niklas Friberg, Oscar Ljungberg, Erik Berglund, David Berglund, Richard Ljungberg, Irina Alafuzoff, Elisabet Englund

**Affiliations:** 1 Department of Immunology, Genetics and Pathology, Uppsala University, Dag Hammarskjölds väg 20, 751 85 Uppsala, Sweden; 2 Department of Clinical Sciences, Division of Oncology and Pathology, Lund University, Sölvegatan 25, 221 85 Lund, Sweden; 3 Department of Pediatrics, Lund University, Lasarettsgatan 40, 221 85 Lund, Sweden; 4 Division of Transplantation Surgery, CLINTEC, Karolinska Institute, Department of Transplantation Surgery, Karolinska University Hospital, Stockholm, Sweden

**Keywords:** Autopsy, Cause of death, Diagnostic error, Referral quality

## Abstract

The use of clinical autopsy has been in decline for many years throughout healthcare systems of developed countries despite studies showing substantial discrepancies between autopsy results and pre-mortal clinical diagnoses. We conducted a study to evaluate over time the use and results of clinical autopsies in Sweden. We reviewed the autopsy reports and autopsy referrals of 2410 adult (age > 17) deceased patients referred to two University hospitals in Sweden during two plus two years, a decade apart. There was a decline in the number of autopsies performed over time, however, mainly in one of the two hospitals. The proportion of autopsy referrals from the emergency department increased from 9 to 16%, while the proportion of referrals from regular hospital wards was almost halved. The autopsies revealed a high prevalence of cardiovascular disease, with myocardial infarction and cerebrovascular lesion found in 40% and 19% of all cases, respectively. In a large proportion of cases (> 30%), significant findings of disease were not anticipated before autopsy, as judged from the referral document and additional data obtained in some but not all cases. In accordance with previous research, our study confirms a declining rate of autopsy even at tertiary, academic hospitals and points out factors possibly involved in the decline.

## Introduction

The clinical autopsy is regarded as the “gold standard” for diagnosis of disease and is an important quality assessment tool in health care. Accurate diagnosis of cause of death and concurrent diseases is important on an individual level for family and relatives but also on a population level for planning of health care and health care research. Clinical autopsy referral rates have been in decline for several decades in Europe as well as in the USA. Reasons for the decline include the clinician’s view that the cause of death is already known, that relatives would oppose autopsy, or that autopsy would not provide additional important information [1–4]. The likelihood of relatives to oppose a proposed autopsy is in turn dependent on the emphasis of the proposition [5, 6]. Furthermore, economic reasons and advancing medical diagnostics may further depreciate the real or apparent need for autopsies [7, 8].

Studies have shown discrepancies between clinical diagnoses and post-mortem autopsy diagnoses in at least 25% of patients, even today [9–11]. A previous assessment of agreement rates between clinical diagnosis and autopsy in a university hospital in Sweden is from 1994 [12]. Since then, changes have occurred not only related to the frequency of autopsies but also to basic regulations. The indications for clinical autopsy in Sweden were extended in 1996 to include investigation of the effects of medical or surgical treatments and investigation of the presence of any disease or injury, in addition to the determination of the cause of death and concurrent diseases [13]. Currently in Sweden, less than 12% of all deceased are assessed post-mortem, half by a clinical and half by a forensic autopsy; the proportion has declined from approximately 50% in the 1970’s [14]. Forensic autopsy is performed in cases of suspected suicide, homicide, death by accident, ongoing drug/alcohol misuse, health care mistreatment, and when death is unexpected or when there is prolonged post-mortem delay, i.e., decomposition of the body, or otherwise difficulty identifying the deceased [13]. Clinical autopsy is performed upon referral from the responsible treating physician (in-hospital patients), from the associated general practitioner or on-call physicians requested for declaring death of a patient (out-of-hospital). In general, the pathologist will, upon referral for autopsy, perform the investigations according to standardized procedures and as far as possible strive to investigate and report on immediate and underlying cause of death as well as on pathological findings of seemingly major importance. Reports of minor findings, not seeming to have been of pivotal importance for either treatable disorders or death, will often also be reported. Fewer autopsies lead to less information being conveyed back to the clinicians making diagnostic and treatment decisions. Therefore, one may fear an increased risk of diagnostic error and missed diagnoses.

In our clinical autopsy practice, we have experienced an impaired quality of autopsy referral texts over the years. This impairment includes insufficient and/or inadequate medical-clinical data and sometimes the mere lack of a clear diagnostic question in the document. While for in-hospital patients, relevant excerpts of the medical record were often attached to the referral document during the time period covered in this study, the only information supplied for out-of hospital patients was that conveyed in the referral sheath. This document has a limited space for text of slightly less than half a regular A4 page. The text could consist of all from singular words to several lengthy sentences. Conceivably, lower quality referrals could lead to lower quality autopsy reports, which could in turn lead to decreased accuracy of feedback on essential unresolved questions and, in turn, an impaired likelihood of future referrals.

We conducted a retrospective study of autopsy reports to evaluate the use and results of clinical autopsies in Sweden. In particular, we wanted to study if there were changes in frequency of autopsy referrals and in referral practice as well as in reported autopsy results over an extended time frame. We also aimed at evaluating, through the reported autopsy findings, the prevalence of important, potentially treatable diseases not known prior to death.

## Materials and methods

### Study population

We studied the autopsy reports of deceased patients referred for clinical autopsy during 4 years in two Swedish University hospitals. The autopsy reports on all consecutive deceased patients referred to the Departments of Pathology at Skåne University Hospital (Lund, Sweden) and Uppsala Akademiska Hospital (Uppsala, Sweden) during 1999, 2000, 2009, and 2010 were analyzed. Exclusion criteria were age < 18 years and incomplete or partial autopsy due to a specific request by the referring physician, the patient or relatives, or due to onward referral for forensic autopsy. Cases were identified retrospectively through the two centers’ electronic autopsy reports and were screened for eligibility.

According to the WHO 10th revision of the International Statistical Classification of Diseases and Related Health Problems (ICD-10) [15] which includes definitions and instructions for the statistics of mortality, the definition of immediate cause of death is the final disease injury or complication directly causing death. The underlying cause of death is the disease, injury, or circumstance that initiated the processes (i.e., intermediate cause of death) which ultimately causes death. All other conditions which to some extent contributed to the fatal outcome are termed contributory causes, however, not part of the main causal sequence leading to death. The underlying cause of death is, according to the WHO, the information to be listed in mortality statistics.

In the present study material, referrals for autopsy often mentioned/suggested a clinical cause of death and the pathologist’s report in general conveyed information on cause of death. Neither the referral nor the report, however, did regularly specify the immediate and underlying cause of death, respectively, even if the one alternative, or both, was included in the referral and report.

### Autopsy report data

The cause of death explicitly mentioned or as evident in the autopsy report was recorded. We did not recode or reclassify (as immediate or underlying) the determined causes of death; the pathologist’s initial diagnoses and terminology were used. The cause of death and significant concurrent diseases (classified as contributory causes) stated in the autopsy report were sorted into 11 predefined categories: aortic rupture/cardiac tamponade, cardiopulmonary failure, cerebrovascular lesion, gastrointestinal hemorrhage, myocardial infarction, pulmonary embolism, pneumonia, sepsis/peritonitis, intestinal ischemia, neoplasm, and other. Brain disease with dementia was recorded as an important disease (contributory cause) in terms of having impact on time of death as well as cause of death, but dementia was rarely considered a cause of death per se. Autopsy findings were compared over the decade interval. Null findings, i.e., situations where the pathologist was unable to judge the cause of death, were also noted.

### Clinical and referral data

The autopsy referral documents were analyzed and patient medical history, length of the referral text, and specific requests by the referring physician (or lack thereof) were noted. The main reasons for referral stated in the autopsy referral documents were noted and sorted into three pre-defined categories: determination of immediate and underlying cause of death, evaluation of a known chronic illness (i.e., usually to evaluate the extent of the disease or the response to a certain medical or surgical therapy), and evaluation of other/predisposing illness. This last category was used where the cause of death was clinically evident (such as hemorrhagic shock by acute gastrointestinal bleeding), but questions regarding unknown, underlying, or predisposing illnesses were specifically stated by the referring clinician. Referrals were sorted by referral text length (> 30 words, 7–30 words, < 7 words, and none).

We noted whether or not the immediate and underlying cause of death stated in the autopsy report was mentioned (suspected or anticipated) in the referral document. We also compared the concurrent diseases identified at autopsy with the diseases mentioned in the referral documents. For each case, we determined whether the autopsy findings (i.e., the immediate, underlying, and contributory cause of death) were unknown, partly known, or fully known before death.

It must be mentioned that during the 4 years during which the individuals in this study underwent an autopsy, the case responsible pathologist would occasionally contact the clinicians in order to obtain more information—this was done with the aim to perform a better analysis, in cases where unacceptably little information was conveyed, so that even the reason for the referral was unclear. This contact was only at times, but most often not noted in the autopsy report. However, in those cases for which the added clinical information included, pertinent facts about major disease or anticipated cause of death, this was added to the report, to be found and reported as known in the present study.

The type of referring clinic was noted and was compared over the decade interval, as were all other items. The cases were sorted per referring clinic into five categories: primary care, emergency department (ED), acute/intensive care unit, medical specialty unit, and surgical specialty unit.

### Data processing

Data from the autopsy records and referral documents were extracted into a standardized form. All data from the two sites were merged regarding findings at autopsy. Categorical data were compared using Pearson’s *χ*^2^ test. A *p* value of < 0.05 was considered statistically significant. All *p* values were two-sided. Data analyses were performed using IBM SPSS Statistics 23.0, Microsoft Excel 365, and R statistical software [16].

### Ethical considerations

An approval from the regional ethical review board was not required for this study according to the Swedish Act concerning the Ethical Review of Research Involving Humans (2003:460) [17].

## Results

### Autopsy referral practices

A total of 2823 reports were reviewed. Four hundred thirteen cases were excluded because of age < 18 years or incomplete autopsy, and the remaining 2410 cases were included in this study. Table 1 shows the baseline data of the study population, as well as the number of referrals from each category of clinical referring unit and the main reasons for referring a deceased patient for autopsy, as judged from the referral text. In many of the numerous cases for which the question about cause of death was raised, it was not possible to judge whether this request connoted immediate or underlying cause of death, or both.

**Table 1:** Autopsy referral data

	1999–2000	2009–2010
Baseline data		
All cases, number (%)	1357	1053
Skåne University Hospital, Lund, *n* (%)	577 (43%)	533 (51%)
Akademiska Hospital, Uppsala, *n* (%)	780 (57%)	520 (49%)
Male, *n* (%)	739/1357 (54%)	666/1053 (63%)
Age, median (Interquartile range)	75 years (65–82)	73 years (63–82)
Referred cases per referring clinic/unit		
Primary care	387 (29%)	335 (32%)
Emergency department	116 (9%)	168 (16%)
Acute/intensive care unit	114 (8%)	138 (13%)
Medical specialty unit	571 (42%)	269 (26%)
Surgical specialty unit	169 (12%)	143 (14%)
Total	1357 (100%)	1053 (100%)
The main pronounced reason for referring for autopsy		
Evaluation of cause of death	972 (72%)	745 (71%)
Evaluation of known chronic illness	181 (13%)	89 (8%)
Evaluation of predisposing illness	169 (12%)	105 (10%)
None noted	35 (3%)	114 (11%)
Total	1357 (100%)	1053 (100%)

Subgroup analyses showed that the cause of death in medical specialty wards often suggested sub-acute or perhaps expected illness, such as cancer or pneumonia. In contrast, the cause of death in the ED more often pointed to an immediate or unexpected death, such as aortic rupture or myocardial infarction (data not shown), hence in the latter cases, the immediate cause of death was apparent or at least suggested clinically.

The extent of the text in the referral document was > 30 words in 1370/2410 (57%) of cases, 7–30 words in 893/2410 (37%), 1–7 words in 102/2410 (4.2%), and no words (blank referral document) in 45/2410 (1.9%). These proportions did not differ significantly between the two study periods.

### Autopsy findings

The cause of death was identified at autopsy in 95.4% of cases (2300/2410). This percentage was similar in both study periods and did not vary with the extent of the autopsy referral document or the main reason for autopsy stated therein as described in Table 1. The major categories of cause of death, as determined at autopsy, were cardiopulmonary failure (23.5%) and myocardial infarction (21.3%), followed by pneumonia (11.4%) (Table 2). Myocardial infarction, as the cause of death, increased from the first to the second period, the others remaining at the same relative level.

**Table 2:** Causes of death at autopsy

	1999–2000		2009–2010			Total	
Cause of death	Number	%	Number	%	*p* value	Number	%
Cardiopulmonary failure	320	23.6%	246	23.4%	0.90	566	23.5%
Myocardial infarct	220	16.2%	293	27.8%	< 0.0001	513	21.3%
Pneumonia	163	12.0%	112	10.6%	0.29	275	11.4%
Neoplasm	140	10.3%	41	3.9%	< 0.0001	181	7.5%
Pulmonary embolism	108	8.0%	56	5.3%	0.011	164	6.8%
Aortic rupture/cardiac tamponade	91	6.7%	63	6.0%	0.47	154	6.4%
Cerebrovascular lesion	82	6.0%	39	3.7%	0.0091	121	5.0%
Sepsis/peritonitis	54	4.0%	51	4.8%	0.30	105	4.4%
Gastrointestinal hemorrhage	26	1.9%	24	2.3%	0.53	50	2.1%
Intestinal ischemia	14	1.0%	19	1.8%	0.11	33	1.4%
Other	77	5.7%	61	5.8%	0.90	138	5.7%
Not identified	62	4.6%	48	4.6%	0.99	110	4.6%
Total	1357	100%	1053	100%		2410	100%

Among all findings, irrespective of judged causality of death, myocardial infarction was the most frequent autopsy finding (39%), followed by cardiopulmonary failure (38%), then by neoplasm (26.9%), and pneumonia (23.2%) (Table 3), the two former findings having increased from the first to the second period.

**Table 3:** Autopsy findings including cause of death

	1999–2000		2009–2010			Total	
	Number	% of *n* = 1357^b^	Number	% of *n* = 1053^b^	*p* value	Number	% of *n* = 2410^b^
Autopsy finding^a^							
Myocardial infarction	476	35.1%	465	44.2%	0.062	941	39.0%
Cardiopulmonary failure	446	32.9%	470	44.6%	0.0018	916	38.0%
Neoplasm	401	29.6%	248	23.6%	< 0.0001	649	26.9%
Pneumonia	294	21.7%	264	25.1%	0.76	558	23.2%
Cerebrovascular lesion	242	17.8%	189	17.9%	0.21	431	17.9%
Pulmonary embolism	160	11.8%	80	7.6%	< 0.0001	240	10.0%
Aortic rupture/cardiac tamponade	138	10.2%	101	9.6%	0.16	239	9.9%
Sepsis/peritonitis	82	6.0%	105	10.0%	0.0083	187	7.8%
Brain disease with dementia	38	2.8%	74	7.0%	< 0.0001	112	4.6%
Gastrointestinal hemorrhage	47	3.5%	45	4.3%	0.67	92	3.8%
Intestinal ischemia	42	3.1%	38	3.6%	0.89	80	3.3%
No result	11	0.8%	5	0.5%	0.21	16	0.7%
Total	2377		2084			4461	
Autopsy findings^c^ known before death							
Yes	279	21%	255	24%	0.032	534	22%
No	428	32%	337	32%	0.81	765	32%
Partially	350	26%	319	30%	0.014	669	28%
No major findings	300	22%	142	13%	< 0.0001	442	18%
Total	1357	100%	1053	100%		2410	100%

^a^Each autopsy often yielded more than one finding

^b^Calculated by the number of autopsy cases, i.e., the prevalence of the illness in this study cohort

^c^All autopsy findings excluding the cause of death

The autopsies often provided information on other concurrent diseases or life-threatening states, i.e., contributing causes. The rates of all pertinent findings are shown in Table 3.

Other findings that were judged to be of lesser importance, yet reported, were identified in 888 additional cases (not shown). These included smaller arterial aneurysms, arteriosclerosis, nephrosclerosis, adenomas of the prostate, and other benign tumors. These did, in general, not seem to be clinically known before autopsy.

Finally, in a considerable number of autopsy reports (23.5%), the declared cause of death was “cardiopulmonary failure.” In many cases, this diagnosis was corroborated by detailed descriptions of various findings and also the exclusion of a number of associated plausible causes, but in others, the connotation of this condition was not fully clear to the present investigators and not fully apparent as from details in the report.

### Concordance of clinical data and autopsy report

The cause of death identified at autopsy was clinically suspected or anticipated in 621/2410 (25.8%) of cases, judging from the referral document and/or the available clinical information. Table 3 describes our further assessment of how many of the pathological conditions found at autopsy that were anticipated clinically. In 32% of all cases, the autopsy report provided information on one or several diseases that were not detailed in the referral document. Table 4 is an account of all significant autopsy findings in those cases.

**Table 4:** Major unanticipated autopsy findings (cause of death not included)

Autopsy finding	Number
Myocardial infarction	221
Cardiopulmonary failure	146
Pneumonia	119
Cerebrovascular lesion	111
Neoplasm	110
Aortic dissection/cardiac tamponade	43
Pulmonary embolism	28
Brain disease with dementia	20
Intestinal ischemia	19
Sepsis/peritonitis	17
Gastrointestinal hemorrhage	16
Other	302
Total	1152

## Discussion

### Autopsy referral practices and referral quality

In this study, we reviewed all complete clinical autopsies of adult patients at two tertiary level hospitals in Sweden for 4 years. Our results were consistent with other data showing declining autopsy rates during the last decades [10, 18, 12]. There was, however, a markedly dissimilar decline in autopsies between the two hospitals, as seen in Table 1. We did not seek to determine the cause of the decline in this study. In Sweden, economic reasons are less likely than elsewhere to discourage autopsy referral since health care, including clinical autopsies, is centrally funded through tax. Even so, autopsies do formally burden the clinical budget, and this may urge clinicians, explicitly or not, to refrain from autopsy referral [8]. The number of forensic autopsies in Sweden has been stable during the last 20 years, reaching an average of 5–6000 cases per year—neither increased nor decreased during the period [19]. A similar stability in number of autopsies was noted in our regional Forensic Medicine Department in the south of Sweden (Forensic Pathology, Lund) where an average of 960 deceased individuals per year come for autopsy (Personal communication, September 2019) .

We found a relative decrease in the proportion of females referred for clinical autopsy, from 46% in the years 1999–2000 to 37% a decade later. This is in agreement with a previous study of similar design, showing the greatest reduction in autopsy rate in females, particularly in women older than 75 years [20].

The pattern of referral changed over time. Referrals from medical wards decreased by more than 50%, accounting for a large proportion of the overall decline in number of autopsies. The reasons for this reduction are not clear. An obvious reason not to refer a patient for clinical autopsy is the assumption that the cause of death is already known. Improvements in diagnostics may thus account for some of the relative decline in the perceived need for autopsy in in-hospital patients.

We hypothesized that autopsy referral document quality has decreased. We analyzed the autopsy referral documents to elucidate the expressed purpose and quality of the referral in terms of comprehensive information (Table 1). As expected, the main reason for referral was to determine the cause of death, only to some extent being detailed as seeking the immediate cause of death. In support of our experience, from the first to the second study period, we found an increasing proportion of low-quality or non-specific referrals. An increasing number of referrals did not specify any purpose, while fewer patients were referred with specific questions regarding a known condition or intervention. According to Swedish law, a main objective of any clinical autopsy is to determine the cause of death [13]. Therefore, one could argue that when no reason is stated (blank referral document), the main reason be determination of the cause of death. However, we believe that a blank referral document does not represent appropriate inter-professional communication in modern health care [21, 22]. Furthermore, this may indicate impaired diagnostic prowess compared to earlier times, or else a lack of interest or time.

We used the number of words in the referral document as a proxy for referral quality but assumed a weak correlation at best between word count and referral quality. In this study, the autopsy referral document consisted of 30 words or less in more than 40% of cases. This cut-off was used because, in our experience, a referral document consisting of 30 words or less, when no other medical information is attached, is unlikely to convey all relevant information about a deceased person’s life and health. Furthermore, a significant number (147) of referral documents consisted of seven words or less. These findings support our hypothesis and naturally leads to the question whether information efforts directed at clinicians could turn around the declining autopsy trend.

### Autopsy findings

The Swedish national cause of death register makes a distinction between the immediate cause of death and the underlying cause of death with the intention that the latter and not the former be included in the registry for each deceased citizen. The underlying cause of death is the preceding or underlying disease or event that started a chain of events that led to death [14]. The World Health Organization (WHO) uses the term “garbage codes” to identify ill-defined conditions registered as the cause of death, meaning symptom and sign conditions [12] and ill-defined cardiovascular diseases. Both the WHO and the Swedish cause of death register act to recode cases with such ill-defined conditions. In the Swedish cause of death register, approximately 20% of all codes for cause of death are not the same as was recorded by the treating physician in the death certificate [14]. Even so, in 1998–2010, approximately 11% of cause of death codes in the Swedish cause of death register were classified as garbage codes by the WHO [23]. In this study, we recorded the primary, i.e., underlying cause of death mentioned in the autopsy report (Table 2), whereas this occasionally also included the immediate cause of death. However, the autopsy protocol sometimes indicated as the cause of death, a condition that would be categorized as a garbage code by the WHO and likewise be insufficient in the Swedish cause of death register. Therefore, our results are not directly comparable to the distribution of cause of death in national and international cause of death registries.

Furthermore, as mentioned above, the autopsy reports often did not specify immediate and underlying cause of death. We were therefore not able to include the distinction in this study. This represents an inherent weakness not only in the clinician-pathologist contact but also in the performed work and reporting of the pathologist.

For changes over time, we noted a significant increase of myocardial infarction, both as the cause of death (from 16 to 28%) and as a concurrent disease finding (from 35 to 44%). At the same time, the relative rates of other manifestations of cardiovascular disease (pulmonary embolism, cerebrovascular lesion, and aortic rupture/cardiac tamponade) tended to decrease over time. The reasons for this pattern was unclear but likely has more to do with selection bias than a real change in disease epidemiology.

We found a relative decrease over time of autopsies where no significant findings were made other than the cause of death itself, from 22% of cases in years 1999–2000 to 13% of cases in years 2009–2010. One explanation could be that with a globally decreasing rate of autopsy, the patients referred for autopsy are those whose death was particularly unexpected or unlikely. Differently, a patient dying with a multitude of known concurrent diseases may have several plausible causes of death. The clinician (faced with a number of real or apparent barriers to autopsy) may feel less need to request an autopsy in such cases than in cases where the deceased patient has no previous medical history [1].

### Concordance of clinical data and autopsy report

Autopsy results are useful for quality assessment and improvement of health care practices. As an estimation of diagnostic errors in medicine, we tried in this study to determine whether the diagnoses found at autopsy had been known to the referring clinician. For comparison, we used the medical information in the referral document, during the study period, the only consistent channel of conveyed clinical information. The percentage of cases whose findings at autopsy were known, suspected, or partially known prior to death increased over time, in spite of the larger proportion of non-specific or low-quality referrals. This is in line with the conclusions drawn in a study of reduced diagnostic errors over time [24]. Still, however, the autopsy reports provided a substantial number of findings presumably unknown before death, in accordance with earlier research [10], something advocating the maintenance of goal-directed autopsies in the quality control of good medical care [25].

Among clinically missed conditions, myocardial infarction was the diagnosis most often first discovered on autopsy, which is not surprising considering the high prevalence of atherosclerotic heart disease in the study population. Also, it is well known that the clinical diagnosis of myocardial infarction or acute coronary syndrome can be difficult to make. More notably, 17% of malignant neoplasms diagnosed at autopsy were not mentioned in the referral document. This is similar to a previous study in which 16% of cancers diagnosed at autopsy were previously unknown [12]. The presence of undiagnosed malignancy may seldom be immediately life-threatening but is an important risk factor for other diseases such as infectious and thromboembolic diseases. It is also likely to be of interest to the patient’s relatives.

### Limitations of this study

As we have shown, the medical information communicated through the referral document was often limited and sometimes lacking, and the pathologist’s access to medical records was only partial. It is therefore likely that not all major health problems seen clinically were known to us and that this led to an overestimation of “unknown” or “missed” diagnoses. At the time of autopsy, however, the pathologist would (as mentioned in “Methods”) request for additional information, at least a minimum of pertinent data as to enable a proper autopsy service—and in cases for which the added clinical information included pertinent facts about major disease or anticipated cause of death (albeit not noted in the original referral), this was added to the report, to be found and reported as known in the present study. This retrieval of additional data took place in a smaller part of cases, the exact number not possible to define retrospectively. Even if this lends to our results a reduced certainty about the claimed discrepancy between clinically known and autopsy recorded data, this is “reality” of everyday autopsy work according to our experience and hence remains a limitation which we would rather describe than try to obscure.

Since we did not have access to the death certificates of the study population, we were unable to directly compare the clinical cause of death to the autopsy report’s cause of death. A review of the full medical record as well as the death certificate for each case would have provided a more accurate estimation of diagnostic discrepancies. Furthermore, it is possible that the difference in autopsy numbers between the two study periods confers a bias, since it implies a selection of the cases referred for autopsy.

At the time of data collection, during years 1999–2010, the referral for autopsy (in both hospitals) required a referral sheath, and for in-hospital deceased patients, this document was often but not always accompanied by parts of the medical records, while often nothing but the referral document was sent for the out-of-hospital deaths.

At present, however, clinical data from all institutions and general practitioners’ clinics are accessible through the medical records’ databases for most of the patients, regardless of where death took place.

No medical (paper) records are attached to the referral. Hence, today, the clinical information is conveyed in a different way, which perhaps could provide a better communication regarding clinical and pathological data and findings, respectively.

This comparison of autopsies in two university hospitals and also of two different decades nevertheless provides a platform for evaluating emerging data and may as such, with all its incompleteness, give valuable information about what new and clinically unknown findings may emanate from an autopsy during these years.

## Conclusions

The clinical autopsy provides information on disease and cause of death which may be unknown to the treating clinician and hence presumably of interest to the treating team and to relatives of the deceased. The diminishing rate of autopsy may impede this important feed-back mechanism. This, in turn, presumably has a negative impact on health care and health care statistics. Efforts are needed to reverse the trend and to specifically point out the benefits of the autopsy as a tool for maintaining good quality in diagnostics and medical health care.

## Data Availability

The datasets generated during and/or analyzed during the current study are available from the corresponding author on reasonable request.

## References

[1] Midelfart J, Aase S (1998). The value of autopsy from a clinical point of view. A survey of 250 general practitioners and hospital clinicians in the county of Sor-Trondelag, Norway. APMIS.

[2] Hull MJ, Nazarian RM, Wheeler AE, Black-Schaffer WS, Mark EJ (2007). Resident physician opinions on autopsy importance and procurement. Hum Pathol.

[3] Burton JL, Underwood J (2007). Clinical, educational, and epidemiological value of autopsy. Lancet (London, England).

[4] Yawson AE, Tette E, Tettey Y (2014). Through the lens of the clinician: autopsy services and utilization in a large teaching hospital in Ghana. BMC Res Notes.

[5] Tsitsikas DA, Brothwell M, Chin Aleong JA, Lister AT (2011). The attitudes of relatives to autopsy: a misconception. J Clin Pathol.

[6] Burton EC, Phillips RS, Covinsky KE, Sands LP, Goldman L, Dawson NV, Connors AF, Landefeld CS (2004). The relation of autopsy rate to physicians' beliefs and recommendations regarding autopsy. Am J Med.

[7] Shojania KG, Burton EC (2008). The vanishing nonforensic autopsy. N Engl J Med.

[8] Nemetz PN, Tanglos E, Sands LP, Fisher WP, Newman WP, Burton EC (2006). Attitudes toward the autopsy--an 8-state survey. MedGenMed.

[9] Winters B, Custer J, Galvagno SM, Colantuoni E, Kapoor SG, Lee H, Goode V, Robinson K, Nakhasi A, Pronovost P, Newman-Toker D (2012). Diagnostic errors in the intensive care unit: a systematic review of autopsy studies. BMJ Qual Saf.

[10] Kuijpers CC, Fronczek J, van de Goot FR, Niessen HW, van Diest PJ, Jiwa M (2014). The value of autopsies in the era of high-tech medicine: discrepant findings persist. J Clin Pathol.

[11] Shojania KG, Burton EC, McDonald KM, Goldman L (2003). Changes in rates of autopsy-detected diagnostic errors over time: a systematic review. JAMA.

[12] Veress B, Alafuzoff I (1994). A retrospective analysis of clinical diagnoses and autopsy findings in 3,042 cases during two different time periods. Human Pathology.

[13] SFS (1995) Lag (1995:832) om obduktion m.m. Swedish Code of Statutes (Svensk Författningssamling (SFS)) (1995:832)

[14] Björkenstam C, Johansson LA (2011). Official statistics of Sweden: Causes of Death 2010.

[15] World Health Organization (1992). The International Statistical Classification of Diseases and Related Health Problems, Tenth revision.

[16] R Core Team (2018) R: a language and environment for statistical computing. R Foundation for Statistical Computing, Vienna, Austria. https://www.R-project.org/

[17] SFS (2003) Lag (2003:460) om etikprövning av forskning som avser människor. Swedish Code of Statutes (Svensk Författningssamling (SFS)) (2003:460)

[18] Chariot P, Witt K, Pautot V, Porcher R, Thomas G, Zafrani ES, Lemaire F (2000). Declining Autopsy Rate in a French Hospital. Arch Pathol Lab Med.

[19] Swedish National Board of Forensic Medicine (2019). Årsredovisning 2018. https://www.rmv.se/wp-content/uploads/Slutlig-ÅR-2018-Rättsmedicinalverket-2019-02-22.pdf.

[20] Lindström P, Janzon L, Sternby NH (1997). Declining autopsy rate in Sweden: a study of causes and consequences in Malmö, Sweden. J Intern Med.

[21] Bion JF, Heffner JE (2004). Challenges in the care of the acutely ill. Lancet (London, England).

[22] Heriot GS, McKelvie P, Pitman AG (2009). Diagnostic errors in patients dying in hospital: radiology’s contribution. J Med Imaging Radiat Oncol.

[23] Mathers C, Stevens G, Retno Mahanani W, Ho J, Ma Fat D, Hogan D (2017) WHO methods and data sources for country-level causes of death 2000-2015. Global Health Estimates Technical Paper. Department of Information, Evidence and Research, WHO, Geneva, Switzerland

[24] Schwanda-Burger S, Moch H, Muntwyler J, Salomon J (2012). Diagnostic errors in the new millennium: a follow-up autopsy study. Modern Pathol.

[25] Goldman L, Sayson R, Robbins S, Cohn LH, Bettman M, Weisberg M (1983). The value of the autopsy in three medical eras. N Engl J Med.

